# Preservation of shrimp quality using dill seed essential oil emulsion in terms of physicochemical, microbiological, and sensory evaluation

**DOI:** 10.1038/s41598-025-18989-6

**Published:** 2025-09-15

**Authors:** Aya Tayel, Faten S. Hassanin, Shimaa N. Edris, Ahmed Hamad, Islam I. Sabeq

**Affiliations:** Department of Food Hygiene and Control, Faculty of Veterinary Medicine, Benha University, Benha, Al-Qalyubia, Egypt

**Keywords:** Shrimp, Essential oil dill, Food safety, Food quality, Natural preservative, Antimicrobials, Sustainability

## Abstract

This study assessed the preservative effects of *Anethum graveolens* essential oil emulsion (*DSEO* emulsion) on the physicochemical, microbiological, and sensory attributes of shrimp (*Litopenaeus vannamei*) during refrigerated storage. Freshly peeled shrimps were divided into five groups: control, butylhydroxytoluene (BHT)-treated (100 ppm), and three *DSEO* emulsion treatments (100, 1000, and 2000 ppm). Samples were examined over 12 days. *DSEO*-3 significantly reduced pH (6.62 ± 0.04), drip loss (0.82 ± 0.07%), and microbial counts (aerobic plate count: 4.11 ± 0.13 log CFU/g; coliforms: <2 log CFU/g) compared to control (pH: 7.21 ± 0.05; APC: 7.64 ± 0.16 log CFU/g). *DSEO*-1 and *DSEO*-3 also reduced cooking loss and improved sensory scores (overall acceptability: 7.4 ± 0.2 vs. control 4.6 ± 0.3 on day 9; *p* < 0.05). *DSEO*-treated groups showed enhanced lightness (L*) and whiteness index values. These findings suggest *DSEO* emulsion, especially at 2000 ppm, is effective as a natural preservative for extending shrimp shelf life and maintaining quality.

## Introduction

Shrimp (*Litopenaeus vannamei*) is a highly valued marine commodity due to its rich nutritional profile and distinct flavor^1^. Its health benefits are attributed to high levels of polyunsaturated fatty acids (PUFA), carotenoids, fat-soluble vitamins, phospholipids, and cholesterol^2^. The quality of shrimp depends on several factors, including the initial condition, species, gender, body composition, time and location of capture, and handling practices^3^. The primary spoilage mechanisms limiting shrimp’s shelf life include oxidation, microbial activity^4^and melanosis formation^5^all of which negatively impact its sensory attributes^6^. The extension of food shelf life is a major focus due to its economic and health implications in the food industry^6^. Freezing and preservatives are commonly employed to maintain shrimp quality, but repeated freezing and thawing cycles can degrade its quality^7^.

Synthetic preservatives such as butylated hydroxytoluene (BHT) are widely used for their antimicrobial and antioxidant properties^8^. However, they are linked to potential toxicity^9^ and are unsuitable for heat processing due to their volatile nature^3^. Due to associated health concerns, some countries have banned their use^10^. Rising concerns about synthetic additives have led to greater interest in natural alternatives, such as plant extracts and essential oils^11^. Essential oils (EOs), derived from aromatic plants, contain bioactive compounds with antimicrobial, antioxidant, antifungal, and insecticidal properties^12^. Numerous studies have demonstrated the effectiveness of EOs in combating spoilage and pathogenic microorganisms in food^13,14^. Due to their natural origin and long history of safe consumption, EOs have gained recognition as food preservatives and align with the current “clean label” movement^15^.

*Anethum graveolens* L., commonly known as dill, is an annual or biennial medicinal plant in the Apiaceae family, widely used in culinary applications, especially in neighboring countries like Turkey^16^. Dill is rich in essential oils, fatty oils, proteins, carbohydrates, fiber, minerals, vitamin A, and niacin^17^. The primary compound in dill seed essential oil is carvone (20–60%), alongside limonene, phellandrene, α-pinene, γ-terpinene, apiole, dill apiole, 1,8-cineole, dihydrocarvone, and p-cymene^18^. Research has demonstrated that dill leaves enhance the shelf life and quality of various foods, including Atlantic bonito fish^19, ^minced meat^20, ^refrigerated beef^21, and ^fresh fish meat^22^.

Given its potent antibacterial and antioxidant properties, *Anethum graveolens* essential oil (*DSEO*) is considered a safer alternative to synthetic preservatives for extending the shelf life of fish products, while also reducing the risk of foodborne pathogens. However, limited studies have assessed the preservative potential of *Anethum graveolens* essential oil in shrimp, which differs structurally and microbiologically from other animal-based foods. Therefore, this study uniquely investigates *DSEO* emulsion effectiveness in preserving *Litopenaeus vannamei*, addressing its specific spoilage mechanisms and quality degradation during refrigerated storage. This study aimed to evaluate the preservative potential of *Anethum graveolens* EO in shrimp (*Litopenaeus vannamei*).

## Materials and methods

### Preparation of dill essential oil

Fresh harvested dill seeds were purchased from a local herbal producer and then delivered for laboratory hydro-distillation extraction. A total of 50 g of powdered dill seeds was hydro-distilled with 750 mL of distilled water using a Clevenger-type apparatus for 3 h at a distillation rate of 1 mL/min. The essential oil yield was 1.6% (v/w) based on the dry weight of the seed. The collected essential oil was transferred into pre-weighed vials using an analytical balance (0.0001 precision) and stored at 4 °C^23^.

### Analysis of chemical compounds in dill essential oil

#### Gas chromatography-mass spectrometry (GC-MS) analysis

The chemical composition of dill seed essential oil was identified using a Trace GC-TSQ mass spectrometer (Thermo Scientific, Austin, TX, USA) equipped with a direct capillary column TG-5MS (30 m × 0.25 mm × 0.25 μm film thickness). The oven temperature was initially set at 50 °C, then increased at a rate of 5 °C/min until reaching 250 °C, where it was maintained for 2 min. Subsequently, the temperature was raised to 300 °C at a rate of 30 °C/min and held for 2 min. The injector and mass spectrometer (MS) transfer line temperatures were set at 270 °C and 260 °C, respectively. Helium gas was used as the carrier at a flow rate of 1 mL/min. The solvent delay was set to 4 min, and a 1 µL sample (diluted) was injected using an Autosampler AS1300 in split mode. Electron ionization (EI) mass spectra were recorded at 70 eV within an m/z range of 50–650 in full scan mode. The ion source temperature was maintained at 200 °C. Chemical components were identified by comparing the obtained mass spectra with reference data from the WILEY 09 and NIST 14 databases^24^. The major volatile compounds identified in *DSEO* using GC-MS included α-phellandrene, d-limonene, carvone, and dill ether, which were selected based on their distinct retention times and mass spectral patterns.

#### Determination of total phenolic content (TPC) in dill essential oil

The Folin–Ciocalteu method was employed to measure the total phenolic content (TPC). A stock solution was prepared by dissolving 1 mL of dill extract in 2 mL of methanol. A 500 µL aliquot of this solution was mixed with 2.5 mL of Folin–Ciocalteu reagent (diluted ten-fold) and 2.5 mL of sodium carbonate solution (75 g/L). The mixture was vortexed for 10 s and then allowed to react for 2 h at 25 °C. The absorbance was measured at 765 nm using a spectrophotometer (Sigma3 30k) against a blank reagent. The calibration curve for gallic acid followed the equation: y = 0.005x + 0.0194 (R² = 0.9977). The gallic acid equivalent (GAE) concentrations were determined by substituting the absorbance values into the equation, and the results were expressed as milligrams of GAE per gram of dried essential oil^25^.

#### Determination of total flavonoid content (TFC) in dill essential oil

The total flavonoid content (TFC) was quantified using a modified aluminum chloride (AlCl3) colorimetric method, as described by^11^. A solution was prepared by dissolving 1 mL of extract in 2 mL of methanol within a 10 mL volumetric flask. Separate solutions of 5% sodium nitrate (NaNO3) and 7% AlCl3 were prepared in a 25 mL volumetric flask using distilled water. A 200 µL aliquot of the extract was mixed with 75 µL of 5% NaNO3 in a sealed glass vial and left in the dark at room temperature for 5 min. Afterward, 1.25 mL of AlCl3 solution and 0.5 mL of sodium hydroxide (NaOH) were added. The solution was then sonicated and incubated for 5 min at room temperature. The absorbance was recorded at 510 nm against a methanol blank. Flavonoid content was determined using a quercetin standard calibration curve, and results were expressed as micrograms of quercetin equivalent (Qu) per gram of dried extract.

#### DPPH free radical-scavenging assay

The antioxidant potential of the dill extract was assessed using the DPPH (2,2-diphenyl-1-picrylhydrazyl) free radical scavenging assay, conducted in triplicate at the Regional Center for Mycology and Biotechnology (RCMB), Al-Azhar University. The method was adapted from^26^ with slight modifications. A freshly prepared 0.004% (w/v) methanolic solution of DPPH was stored at 10 °C in a dark environment. A methanolic solution of the test extract was prepared, and a 40 µL aliquot was added to 3 mL of the DPPH solution. Absorbance measurements were taken immediately using a UV-visible spectrophotometer (Milton Roy, Spectronic 1201). The decrease in absorbance at 515 nm was continuously recorded at 1-minute intervals until it stabilized (approximately 16 min). Control measurements were performed using a DPPH solution without antioxidants, and ascorbic acid was used as a reference compound. All analyses were conducted in triplicate. The percentage inhibition (PI) of DPPH radicals was calculated using the formula:

$${\text{PI}}=\left[ {\left( {\left( {{\text{AC}} - {\text{AT}}} \right)/{\text{AC}}} \right) \times 100} \right]$$

Where AC = Absorbance of the control at t = 0 min and AT = absorbance of the sample + DPPH at t = 16 min.

### Shrimp sample collection and Preparation

**Fig. 1:**
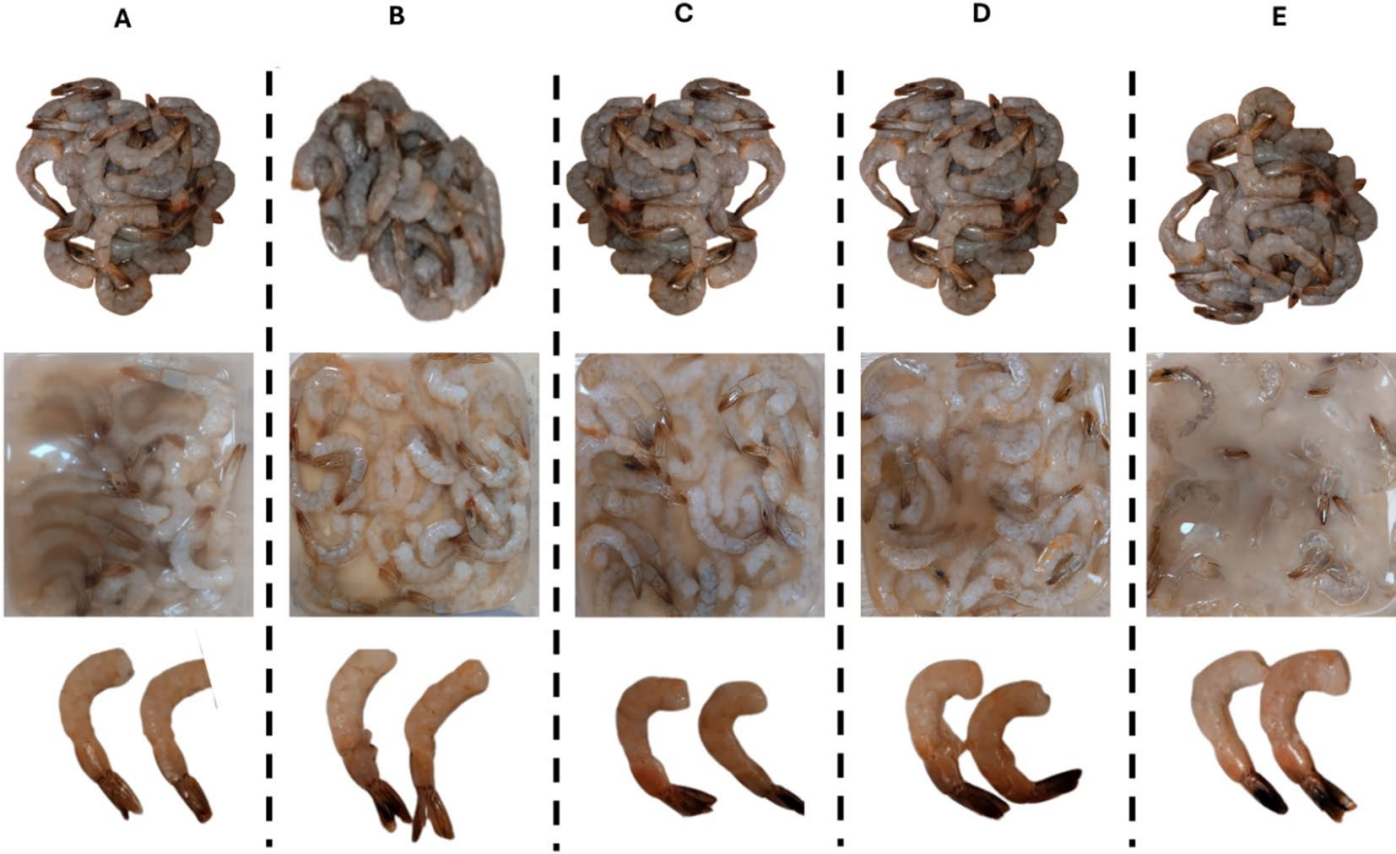
Preparation and treatment of shrimp samples with BHT and dill seed essential oil (DSEO) at three concentrations. (**A**) Control group; (**B**) BHT-treated group; (**C**) DSEO (100 ppm); (**D**) DSEO (1000 ppm); (**E**) DSEO (2000 ppm).

This study was conducted on freshly peeled shrimp (*Litopenaeus vannamei*) with telson, from a local seafood restaurant in Banha City, Egypt, between October and November 2024. Fresh shrimp (5 kg) visually inspected for freshness (firm texture, bright color, and no off-odors) were selected for the study were transported in ice (0 °C) to the laboratory, and shrimp (12 ± 3 g each) were randomly divided into five groups (65 shrimp/group). The control shrimp were dipped in sterile distilled water. The control group was treated with 100 ppm butylated hydroxytoluene (BHT). For the other three groups, shrimp were treated with different concentrations of Dill SEO: 100 ppm (*DSEO*-1), 1000 ppm (*DSEO*-2), and 2000 ppm (*DSEO*-3). Control samples were dipped in sterile DW, whereas BHT-treated shrimp were dipped in BHT solution (100 ppm, w/v). The dill seed essential oil (*DSEO*) emulsion was prepared by dissolving *DSEO* in sterile distilled water containing Tween 80 (0.8%, w/v) as an emulsifier. The mixture was homogenized using a high-speed homogenizer at 1000 rpm for 10 min to form a stable emulsion before being used for shrimp treatment. The samples were immersed in 1000 mL of either the negative or positive control (BHA) and three concentrations of Dill emulsion for 30 min at room temperature (22 ± 1 °C) (Fig. 1).

The samples were removed from the dipping suspensions and left to leak through a sterile sieve for 15 min. Samples from each group were distributed into two zipper bags (5 shrimps/bag), spread over five checking points (total 50 shrimps/group: 5 shrimps× 2 trials ×5 intervals), and incubated in a programmable cooling incubator at 2.5 ± 0.5 (Binder, Germany) which falls within the standard refrigerated storage range for seafood (0–4 °C), as defined by Codex and EU guidelines. The samples were then examined for physicochemical characteristics, microbial count, and sensory evaluation over 12 days at specified checkpoints (1, 3,6, 9, and 12 days post-treatment).

### Physicochemical analysis of peeled shrimp

The physicochemical characteristics of peeled shrimp were evaluated following previously established methods^27^with slight modifications.

#### pH measurement

The pH of shrimp samples was measured using a calibrated digital pH meter (Jenway 3510, UK) equipped with a glass electrode. For each sample, 10 g of minced shrimp was homogenized with 90 mL of distilled water using a stomacher for 1 min. Calibration of the pH meter was conducted at room temperature using buffer solutions with pH values of 4, 7, and 10, along with a temperature metal probe. The pH of the resulting slurry was measured at room temperature, and the electrode was rinsed with distilled water and recalibrated as needed between samples^28^.

#### Water-holding capacity (WHC) Estimation

The WHC of peeled shrimp was assessed using the filter paper press method (FPPM). A sample weighing between 0.2 and 0.5 g was placed on Whatman No. 1 filter paper and subjected to a 5 kg weight for 30 s. The WHC was calculated as the percentage of water retained after deducting the released moisture from the initial sample weight^28^.

#### Purge loss measurement

To evaluate purge loss, two cylindrical shrimp samples (12 ± 3 g each) were used. The percentage of weight loss from the initial recorded weight at the start of refrigerated storage was determined at different intervals (0, 3, 6, 9, and 12 days post-treatment)^29^.

#### Cooking loss (CL) assessment

Following purge loss estimation, two shrimp samples were selected to determine cooking loss (CL). The peeled shrimp (cylindrical shape, 12 ± 3 g each) were sealed in thin-walled, heat-resistant plastic bags and cooked at 80 °C for 10 min in a water bath. Afterward, the samples were cooled to room temperature using tap water, followed by chilling in an ice bath at 5 °C. The shrimp were then dried and reweighed. Cooking loss was calculated as the percentage difference between the raw and cooked sample weights^30^.

#### Warner–Bratzler shear force (WBSF) analysis

Shear force analysis of cooked shrimp was conducted using a 3343 Universal Testing Device Mono column (Instron, Norwood, MA, USA). The shrimp cores were sheared perpendicular to the muscle fibers. The Warner–Bratzler shear force (WBSF) was expressed in kilograms of force (KGF) and determined as the average of six cores from each sample^31^.

#### Color estimation

Three colors, L*, a*, and b*, were measured in raw M. longissimus using a chromometer CR-410 (Konica Minolta Sensing Inc., Osaka, Japan). The Chroma Metre was set to L*, a*, b* color space and illuminant D65, with a 2° observer angle and 8.0 mm aperture size with a closed cone. The chromameter was calibrated with a standardized white tile before measuring the cut surface of the peeled shrimp after 30 min of blooming. These color values were then used to estimate color saturation as (Hue angle (h˚) = arctg b */a *) and color intensity as (C = (a*^2 + b*^2) ^0.5). The average of the six measurements was calculated for each group. Higher chroma levels indicate an increased saturation of the primary hue of the sample. In contrast, higher hue angle (or color intensity) values indicate a lower quantity of meat^32^and the total color difference (ΔE), which indicates the amount of color difference between shrimp before and after storage was calculated as follows (ΔE)$\:={\left[\left({L}^{*}-{{L}^{*}}_{0}\right)2+\left({a}^{*\:}-{{a}^{*\:}}_{0}\right)\:2+\left({b}^{*}-{{b}^{*}}_{0}\right)2\right]}^{1/2}$^33^.

The whiteness index (WI) combines lightness, yellow, and blue into a single term to estimate the whiteness degree^34^. The WI was determined according to (Qian et al. 2013)

$$\:WI=100-\sqrt{{{\left(100-L*\right)}^{2}+a*}^{2}+{b*}^{2}}.$$

The yellowness indices (YI) is the grade by which the sample surface is different from the ideal white in terms of yellowness^19^ YI = 142.86*b/L.

The white-leg shrimp browning function was assessed by the Browning Index (BI) $\:=\frac{100}{0.17}(\frac{a*+1.75L*}{5.645L*+a*-0.012b*}-0.31)$^35^.

### Microbial analysis

The microbial quality of peeled shrimp was monitored over 12 days (days 0, 3, 6, 9, and 12) to assess aerobic plate count (APC), coliform count, lactic acid bacteria count, and staphylococcal count. All analyses were conducted in a binder incubator (BINDER GmbH, Tuttlingen, Germany) at a controlled temperature of 2.5 ± 0.5 °C.

#### Aerobic plate count (APC) determination

The aerobic plate count (APC) in shrimp samples was determined using the method applied in ground beef products^36^. A 10% homogenate was prepared by aseptically weighing 10 g of each sample and blending it with 90 mL of sterile distilled water using a Stomacher 400R (Seward, UK). The homogenized samples underwent tenfold serial dilution in sterile distilled water. A 1 mL aliquot from each dilution was plated on two sterile plate count agar plates (Condalab, Spain). The plates were incubated at 37 °C for 24 h, and colony counts were recorded as log CFU/g.

#### Coliform count determination

To determine the coliform count, 1 mL from the serially diluted shrimp homogenates was inoculated onto violet red bile agar (Himedia Laboratories, India) and incubated at 37 °C for 24 h for enumeration^37^. The results were expressed as log CFU/g.

#### Staphylococcus count determination

The enumeration of *Staphylococcus* in peeled shrimp was conducted by surface plating on Baird Parker agar (Oxoid, United Kingdom), following a method previously used for milk^38^. A 1 mL aliquot from each serial dilution was spread over the agar using a sterile disposable spreader. After allowing the inoculum to be absorbed (10 min to 1 h), the plates were inverted and incubated at 37 °C for 48 h^39^. Colony counts were recorded as log CFU/g.

#### Lactic acid bacteria (LAB) count

The enumeration of LAB was performed using Man, Rogosa, and Sharpe (MRS) agar (HiMedia, USA). Serially diluted shrimp homogenates were cultured by the spread plate technique and incubated anaerobically in a Gas Pak Jar at 30 °C for 72 h^36^. Colony counts were reported as log CFU/g.

### Sensory evaluation

A panel of ten trained evaluators conducted a sensory assessment of raw-peeled shrimp. Training continued until panelists demonstrated consistency, with scores differing by no more than one unit from the group mean. Representative samples were served on porcelain plates without identifying information, and each treatment was analyzed in triplicate. Panelists rated freshness using a nine-point hedonic scale, scoring attributes between 1 and 9 based on sensory quality criteria. Evaluations included color, odor, texture, and overall appearance. Shrimp samples were categorized from “extremely dislike” (score 1) to “like extremely” (score 9), based on overall acceptability. No additional preparation was performed on the samples before evaluation^40^.

### Statistical analysis

Data analysis was conducted using SPSS Version 22 (SPSS Inc., Chicago, IL, USA). The effects of antimicrobial dipping treatments, storage duration (1, 3, 6, 9, and 12 days), and their interactions on physicochemical, microbiological, and antioxidant properties of peeled shrimp were analyzed using general linear models (GLM). In this model, fillet samples were considered random variables, while antimicrobial treatment and storage time were fixed factors. Mean values and standard errors were reported. Tukey’s b multiple comparison test was employed to determine the differences between antimicrobial treatments and the control group, as well as to compare means across different storage time points within the same group. A significance level of *P* < 0.05 was used to identify statistically significant differences. Principal Component Analysis (PCA) was performed using Scikit-learn (Python 3.9) to evaluate the impact of treatments and storage time on shrimp quality. Before PCA, the data were standardized using z-score normalization. The analysis included microbiological, physicochemical, and color attributes, with dimensionality reduction to two principal components (PC1 and PC2). The PCA biplot was generated using Matplotlib, where treatment groups and storage durations were visually represented based on their principal component scores. Quality parameters were illustrated as directional vectors, indicating their contribution to the overall variance.

## Results

**Fig. 2:**
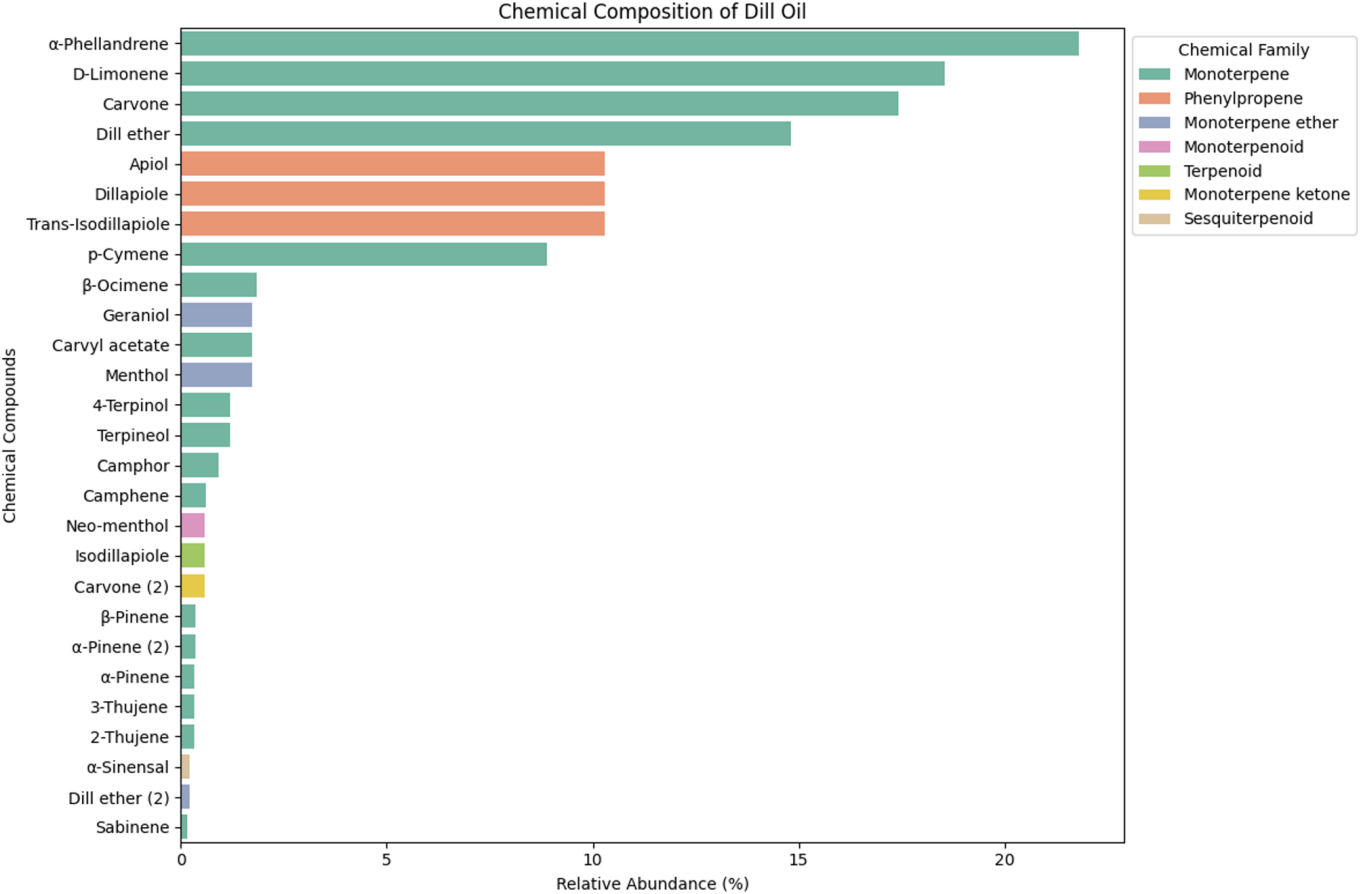
Chemical Composition of dill seed essential oil using GC-MS.

Gas chromatography-mass spectrometry (GC/MS) analysis of dill seed essential oil provided a chemical profile of its constituents, offering insights into its potential functional and therapeutic applications (Fig. 2). GC-MS analysis revealed the presence of multiple bioactive compounds, each with distinct retention times and mass spectral patterns. The profile indicated that monoterpenes dominated the volatile fraction and accounted for the majority of the identified compounds. The presence of monoterpenes such as α-phellandrene (21.81%), d-limonene (18.54%), carvone (17.42%), and dill ether (14.82%) suggests their potential role in flavor enhancement and biological activity. These compounds contribute to the characteristic aroma and taste of dill essential oils and their antimicrobial and antifungal properties.

The total phenolic content (TPC) and total flavonoid content (TFC) in dill seed essential oil were quantified using the Folin-Ciocalteu colorimetric method and modified aluminum chloride (AlCl3) colorimetric method, respectively (Table 1). These assessments provided insights into the bioactive compounds present in the extract, contributing to their potential antioxidant and therapeutic properties.

The phenolic content of dill seed essential oil was 81.5 mg gallic acid equivalent (GAE) per gram of extract (Fig. 2). This value indicates a substantial concentration of phenolics recognized for their antioxidant activities, suggesting that dill essential oils may contribute to mitigating oxidative stress in biological systems. Additionally, dill seed essential oil had a flavonoid content of 49.5 mg quercetin equivalent (QuE) per gram of extract (Table 1). Flavonoids are notable for their diverse pharmacological properties, including their anti-inflammatory and antioxidant effects.

**Table 1:** Total phenolic and flavonoid concentration of dill essential oil.

Tested oil	Phenolic concentration mg(gal)/gm	Flavonoid concentration. mg(QuE)/gm
Dill essential oil	81.5	49.5

**Fig. 3:**
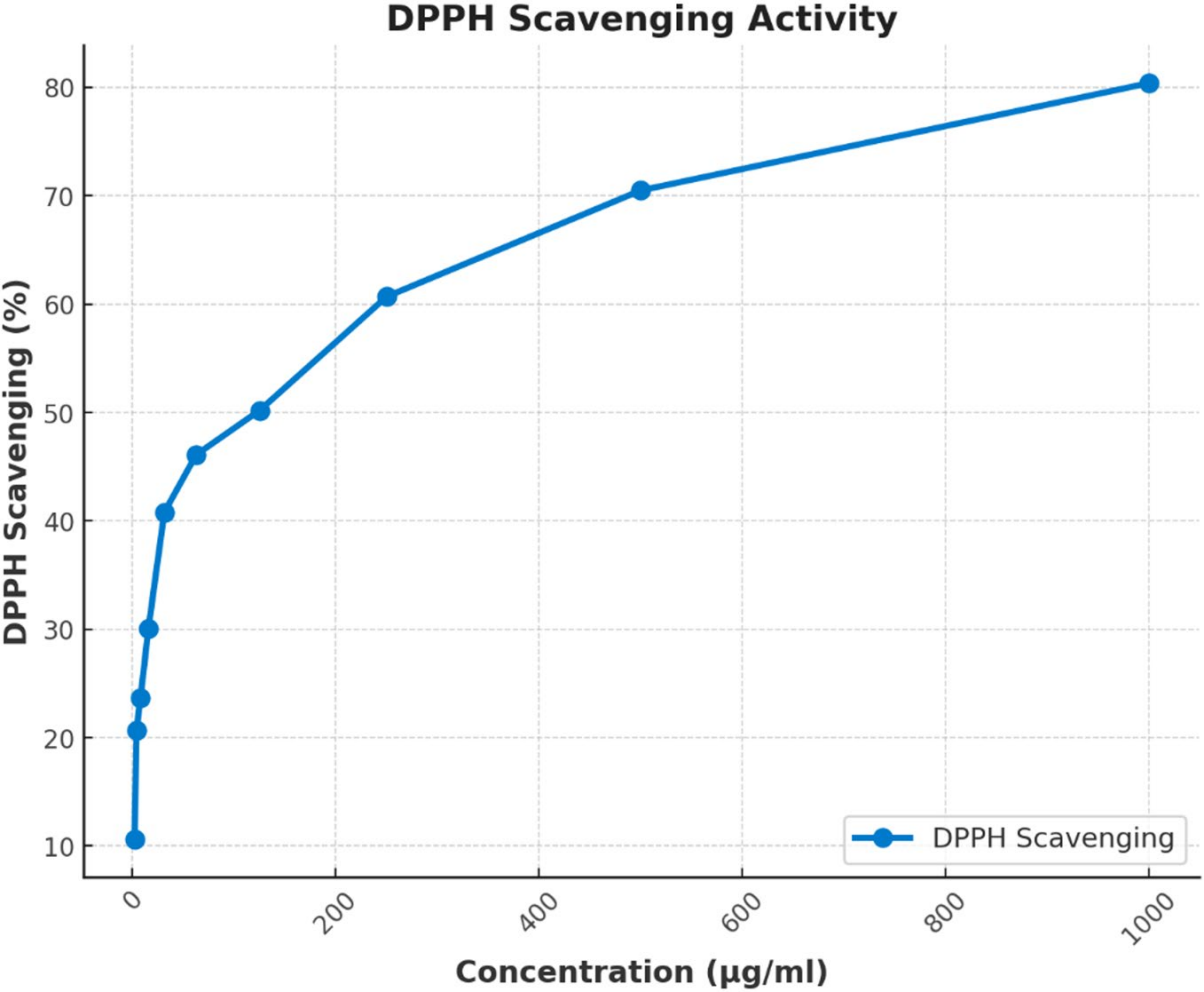
DDPH scavenging % of dill essential oil.

The antioxidant activity of dill seed essential oil was evaluated using the DPPH (2,2-diphenyl-1-picrylhydrazyl) radical scavenging assay (Fig. 3). Dill seed essential oil showed dose-dependent antioxidant activity, with DPPH scavenging percentages ranging from 17.6% at 1.95 µg/ml to 80.4% at 1000 µg/ml. The IC50 value, representing the concentration required to inhibit 50% of DPPH radicals, was 48.3 ± 0.9 µg/ml. This indicates moderate antioxidant potency, suggesting that dill seed essential oil contains compounds capable of donating electrons or hydrogen atoms to neutralize free radicals.

**Fig. 4:**
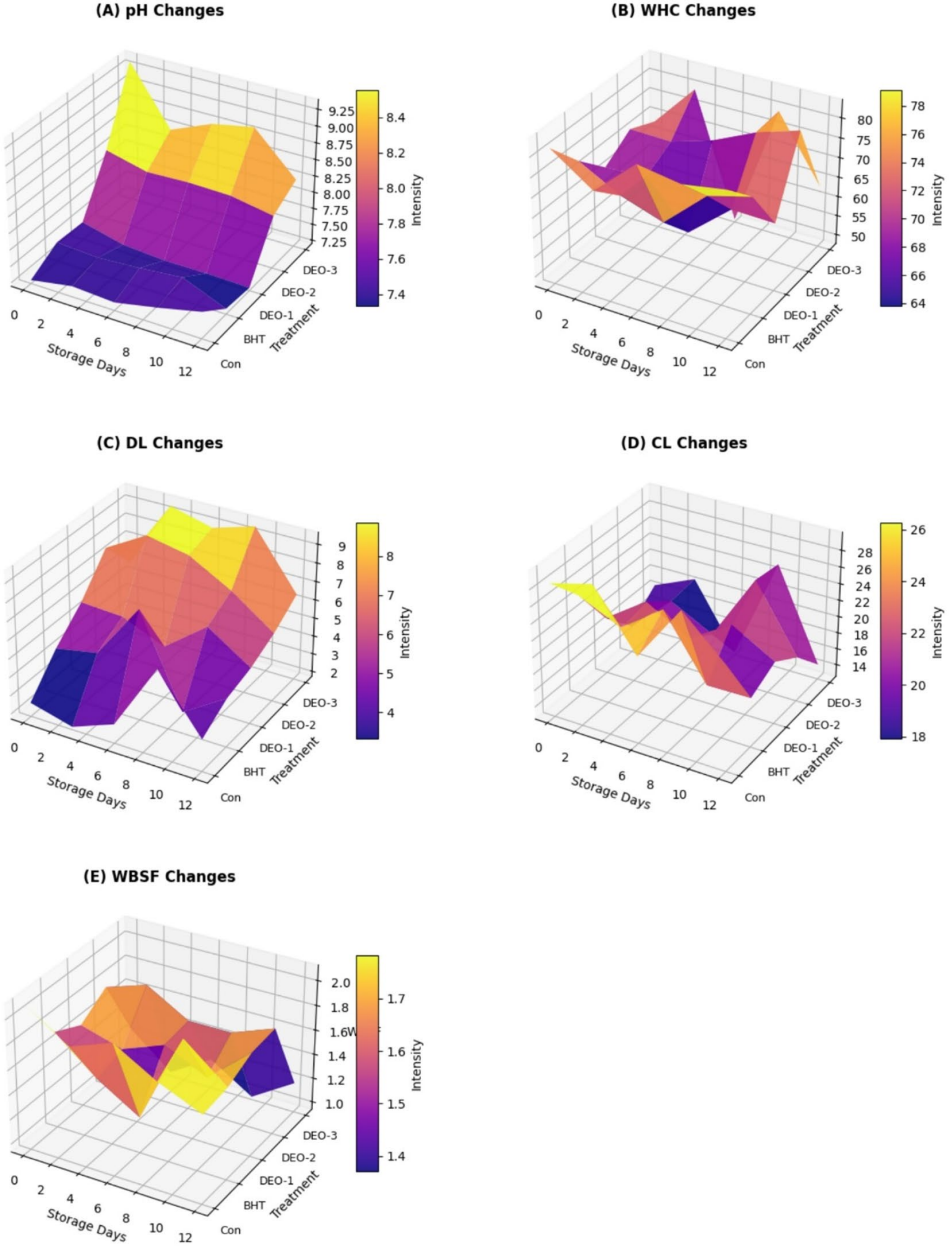
Changes in physicochemical parameters over time in shrimp meat subjected to different dill seed essential oils and BHT treatments compared to the control group after 12 days of refrigeration storage. Figure generated by Python (Matplotlib and NumPy libraries).

The physicochemical parameters of shrimp were significantly influenced by dill oil application (*P* < 0.05), except for water-holding capacity (WHC) and drip loss (DL) (*P* > 0.05). The pH value of dill oil-3 (*DSEO*-3) was significantly higher than that of the control and other treated groups on the first day post-treatment (*P* < 0.05) (Fig. 4). The control group exhibited an upward increase in pH value during storage (*P* < 0.05), with *DSEO*-3 demonstrating the lowest pH value compared to the other treated groups (*P* < 0.05). There was a statistically insignificant increase (*P* > 0.05) in WHC values across all groups, except *DSEO*-3, which showed a significantly higher WHC value on day 9 compared to the BHT and control groups (*P* < 0.05). The WHC values decreased on day 12 post-treatment, except for the BHT and *DSEO*-1 groups (*P* > 0.05). The control group exhibited an upward trend in drip loss (DL) and pH throughout storage. All treated groups showed a negligible decline (*P* > 0.05) in DL value, except for *DSEO*-2, which had the lowest DL value compared to *DSEO*-1 (*P* < 0.05). *DSEO*-3 had the lowest DL value on days 6 and 12 following the treatment (*P* > 0.05). No discernible trend was observed in cooking loss (CL) across groups. On day 3 post-treatment, *DSEO*-2 and *DSEO*-3 exhibited the lowest CL values compared to *DSEO*-1 (*P* < 0.05). The CL values of *DSEO*-1 and *DSEO*-3 were significantly lower than those of *DSEO*-2 on days 9 and 12 (*P* < 0.05). Regarding Warner Bratzler Shear Force (WBSF), on the initial storage day, the control and *DSEO*-1 demonstrated the greatest values of 2.10 and 2.00, respectively, denoting a firmer texture. BHT, *DSEO*-2, and *DSEO*-3 exhibited markedly reduced WBSF values, indicating an initial softening effect relative to that of the control and *DSEO*-1 (*P* < 0.05). The control group demonstrated a decreasing WBSF trend over time, reaching its minimum at D-6 (1.61), followed by a minor increase at D-9 (1.76), and lowering again on day 12 (1.25). The BHT-treated group had a downward trend, followed by a modest increase, peaking at day 9 (1.67), and declining again at day 12 (0.96). This indicates that BHT aids in maintaining the structural integrity, postponing the softening process. Samples treated with *DSEO* 1 showed a decreasing pattern, peaking on the third day of storage (1.23), followed by an increase in mid-storage, and a decrease on the last day (1.18). *DSEO*-2 had an upward trajectory, peaking on day 3 at 1.69, and then declining on days 6 and 12 to 1.48 and 0.96, respectively. The most notable pattern was in the *DSEO* 3-treated group, showing an increasing WBSF trend, peaking at D-9 (1.80) before decreasing at D-12 (1.18). This indicates that *DSEO* 3 initially improved muscular stiffness, but this effect was reduced after extended storage.

**Fig. 5:**
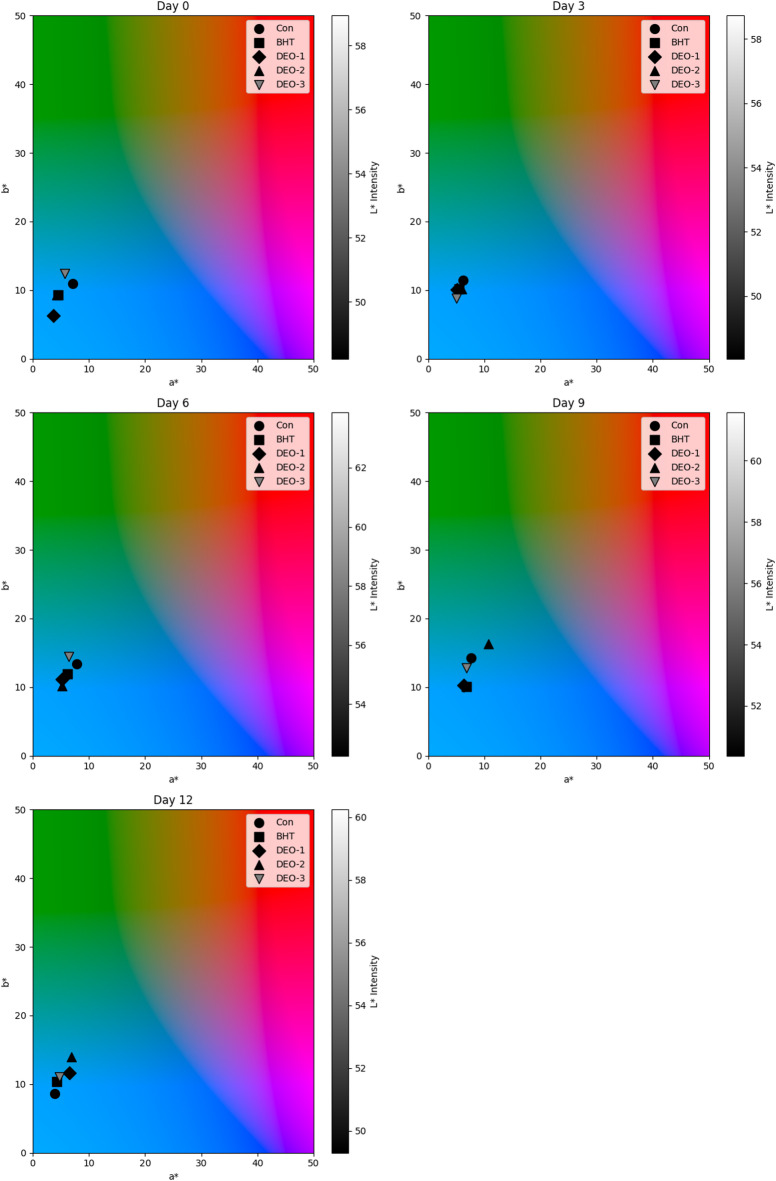
Changes in color parameters of shrimp meat samples subjected to different treatments over a 12-day storage period. a* (Red-Green Axis): measures the degree of redness (+ a*) or greenness (-a*), primarily influenced by myoglobin oxidation; b* (Yellow-Blue Axis): yellowness (+ b*) or blueness (-b*), often associated with lipid oxidation and pigment degradation. L* (Lightness Intensity): Depicted by the color gradient, with darker shades representing lower L* values and lighter shades indicating higher L* values.

In terms of color indices, the influence of dill seed essential oil on color indices of shrimp was indicated on Figure (5). Regarding the lightness (L*) index, the control and BHT-treated groups exhibited a lower L* index on the first day post-treatment, whereas all *DSEO*-treated groups showed higher L* values (*P* < 0.05). On the third day, the control group exhibited a greater L* value than the BHT- and *DSEO*-treated groups (*P* < 0.05). On the sixth day, *DSEO*-3 exhibited a greater L* value than the other groups (*P* < 0.05). On the ninth day, the *DSEO*-2 treated groups had the highest L* value, while the BHT-treated groups had the lowest. On the last day, the BHT-treated group had a greater L* value than the other groups (*P* < 0.05).

Concerning the redness (a*) index, all treated groups demonstrated a reduced a* value relative to the control on the D-Zero post-treatment (*P* < 0.05). On the third day, both *DSEO*-1 and *DSEO*-3 exhibited reduced a* values compared to the other groups (*P* < 0.05). On Day 6, all treated groups showed a reduced a* value compared with the control group (*P* < 0.05). On days 9 and 12, *DSEO*-2 had the highest a* value compared with the other groups (*P* < 0.05).

Regarding the yellowness (b*) index, on the first day, the *DSEO*-3 group demonstrated a significantly elevated b* value relative to the other groups (*P* < 0.05). On Day 3, *DSEO*-3 displayed a reduced b* value compared with the control group (*P* < 0.05). On Day 6, *DSEO*-3 exhibited a higher b* value than the other groups (*P* < 0.05). On Days 9 and 12, *DSEO*-2 had the highest b* value compared with the other groups (*P* < 0.05).

On day zero, all treated groups, except *DSEO*-1, showed a substantial increase in hue value compared to the control group (*P* < 0.05). The *DSEO*-1 hue was the highest on Day-3. *DSEO*-3 had the highest hue values on days 6 and 9 (*P* < 0.05). On the final day, the BHT-treated groups had a greater hue value than the other groups (*P* < 0.05). The chroma index for *DSEO*-3 and the control group was higher than that for the other treatment groups (*P* < 0.05). The control chroma value was significantly higher in the BHT- and *DSEO*-treated groups (*P* < 0.05) on the third day post-treatment. Similarly, the control and *DSEO*-3 groups exhibited comparable values on day-zero post-treatment (*P* < 0.05). On days 9 and 12, the *DSEO*-2 treated groups exhibited superior chroma values compared with the control and other treated groups (*P* < 0.05), which was consistent with the results of the redness and yellowness indices (Fig. 5).

**Fig. 6:**
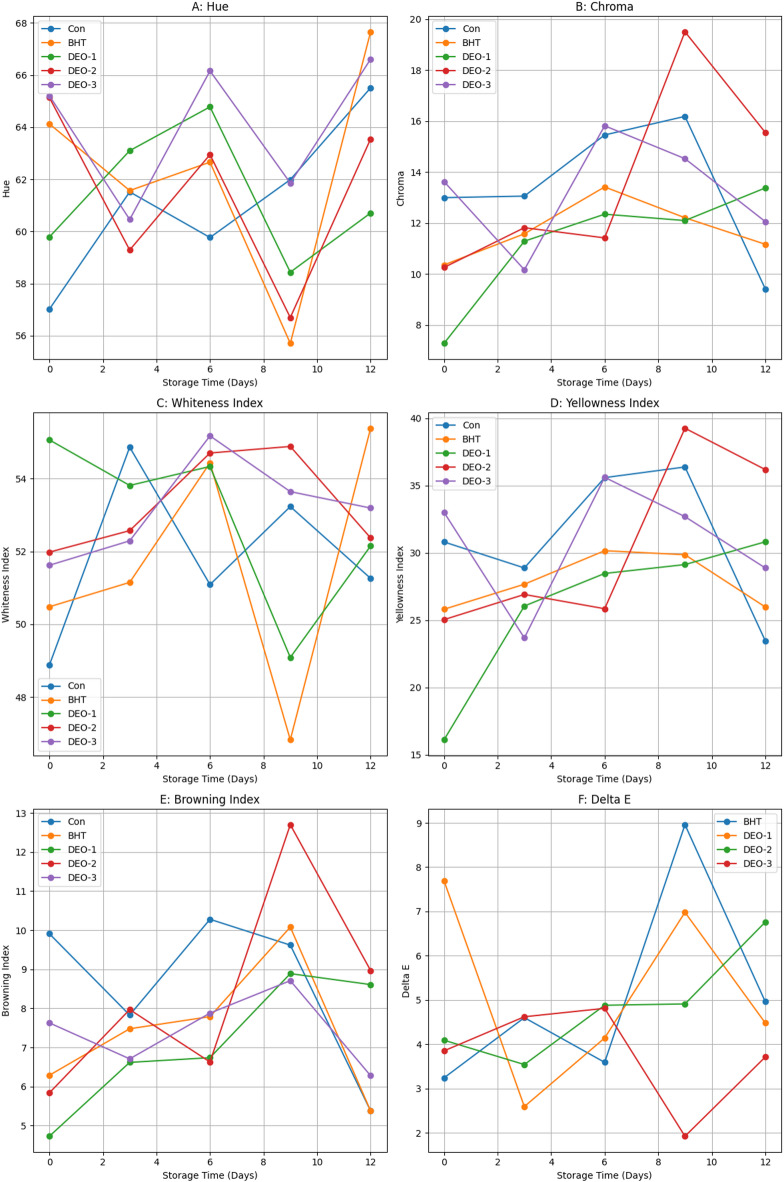
Changes in color stability parameters of shrimp meat during storage under different dill seed essential oil treatments.

The whiteness index (WI) increased in all fortified groups, particularly in those with BHT and *DSEO*. *DSEO*-1 showed the highest value compared to that of the control on the first day of storage (*P* < 0.05) (Fig. 6). On day three, the BHT group had a lower WI than that of the control group (*P* < 0.05). By day six, the BHT and *DSEO* groups had greater WI values than the control group (*P* < 0.05). *DSEO*-2 had a higher WI than that of the control on day nine (*P* < 0.05). On day 12, the BHT and *DSEO* groups showed superior WI values compared with the control, with BHT being the highest (*P* < 0.05). From day one, *DSEO*-2 and *DSEO*-3 increased throughout storage, peaking on days 6, 9, and 12 (*P* < 0.05). *DSEO*-1 showed a fluctuating trend, peaking on days one and six (*P* < 0.05). The yellowness index (YI), BHT, and *DSEO* groups, except for *DSEO*-3, had lower values than the control on day one (*P* < 0.05) (Fig. 6). No significant differences were observed on day three (*P* > 0.05). On day six, all fortified groups, except for *DSEO*-3, had lower YI values than the control group (*P* < 0.05). On days 9 and 12, *DSEO*-2 showed a greater YI increase than the control (*P* < 0.05). The control, BHT, *DSEO*-1, and *DSEO*-2 groups showed an increasing and declining YI trend, peaking on days 6 and 9 for the control and BHT, and days 9 and 12 for *DSEO*-1 and 2 (*P* < 0.05). *DSEO*-3 showed no clear trend, peaking on days 6 and 9 (*P* < 0.05). For browning index (BI), BHT, and *DSEO* groups had significantly lower values than the control on day one (*P* < 0.05). On the third day post-treatment, no significant differences in BI values were observed among the groups (*P* > 0.05) (Fig. 6). On the sixth day, the BHT and all *DSEO*-fortified groups showed a decline in BI values relative to the control group (*P* < 0.05). On days 9 and 12, the *DSEO*-2 fortified group showed a significant increase in BI value relative to the control group (*P* < 0.05). During extended storage, the BHT and *DSEO*-1 groups showed a downward trend in BI value, followed by an upward trend and then downward, peaking on day 9 for BHT and days 9 and 12 for *DSEO*-1 relative to the initial storage-point (*P* < 0.05). The control and *DSEO*-3 groups showed a decreasing trend, with peaks on days 1, 6, and 9 for the control and day 9 for the *DSEO*-3 group (*P* < 0.05). *DSEO*-2’s BI value showed no specific trend, peaking on day 9 (*P* < 0.05). For delta E (∆E), the total color difference values showed no significant differences among treatments at the same intervals (*P* > 0.05), except on the initial day and days 9 and 12, with the highest values on days 1, 9, and 12 for *DSEO*-1, control, and *DSEO*-2, respectively (Fig. 6). The ∆E values of the BHT and all *DSEO*-fortified groups showed no clear trend throughout the storage period compared with the first storage-point.

**Fig. 7:**
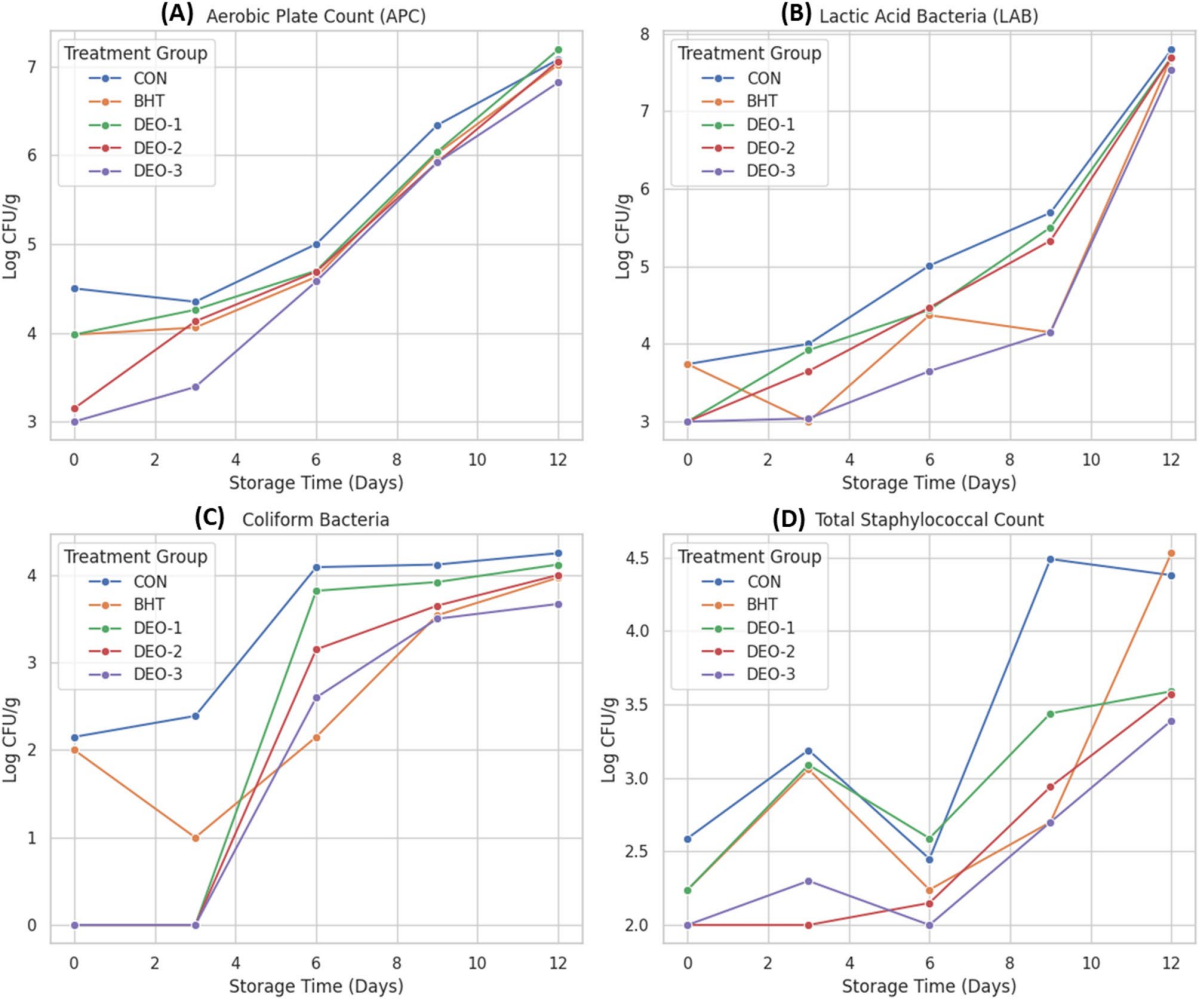
Microbial analysis of shrimp meat stored under refrigeration (2.5 ± 0.5 °C) for 12 days after treatment with Dill seed essential oil emulsion (*DSEO*) at three concentrations (*DSEO* 1, *DSEO* 2, and *DSEO* 3), compared with the Control and BHT. (**A**) Aerobic Plate Count (APC), (**B**) *Lactic Acid Bacteria* (LAB) count, (**C**) coliform count, and (**D**) Total Staphylococcal Count.

The impact of BHT and/or *DSEO* on APC of treated shrimp groups compared to control is shown in Fig. 7. On the first day, *DSEO*-3 exhibited a lower APC count than that of the control and other groups (*p* < 0.05). The APC count decreased in the fortified-*DSEO* groups, particularly *DSEO*-2 and *DSEO*-3, on the ninth day of storage (*p* < 0.05) compared to that in the control and other groups, despite an upward trend during storage (*p* < 0.05). LAB were not substantially influenced by BHT and/or *DSEO* for up to 12 d, with two exceptions. On the sixth and ninth days, the BHT-treated and *DSEO*-3 treated groups showed reduced LAB counts relative to the control and other groups (*p* < 0.05), as shown in Fig. 1B. The coliform count was reduced (below 2 log) in all *DSEO*-treated groups on the first and third days compared to the BHT-treated and control groups (*p* < 0.05). *DSEO*-3 treated groups had reduced coliform counts on days 6 and 9 compared with the other groups (*p* < 0.05). The BHT-treated groups showed reduced coliform counts on day 9 compared with the other groups (*p* < 0.05), as illustrated in Fig. 1C. The overall staphylococcal count was diminished by *DSEO*, particularly *DSEO*-2 and *DSEO*-3, on the third day (*p* < 0.05) compared to the control and other groups. On the ninth day, the *DSEO*-3- and BHT-treated groups showed a significant decrease in total staphylococcal count compared to the control and other groups (*p* < 0.05).

**Fig. 8:**
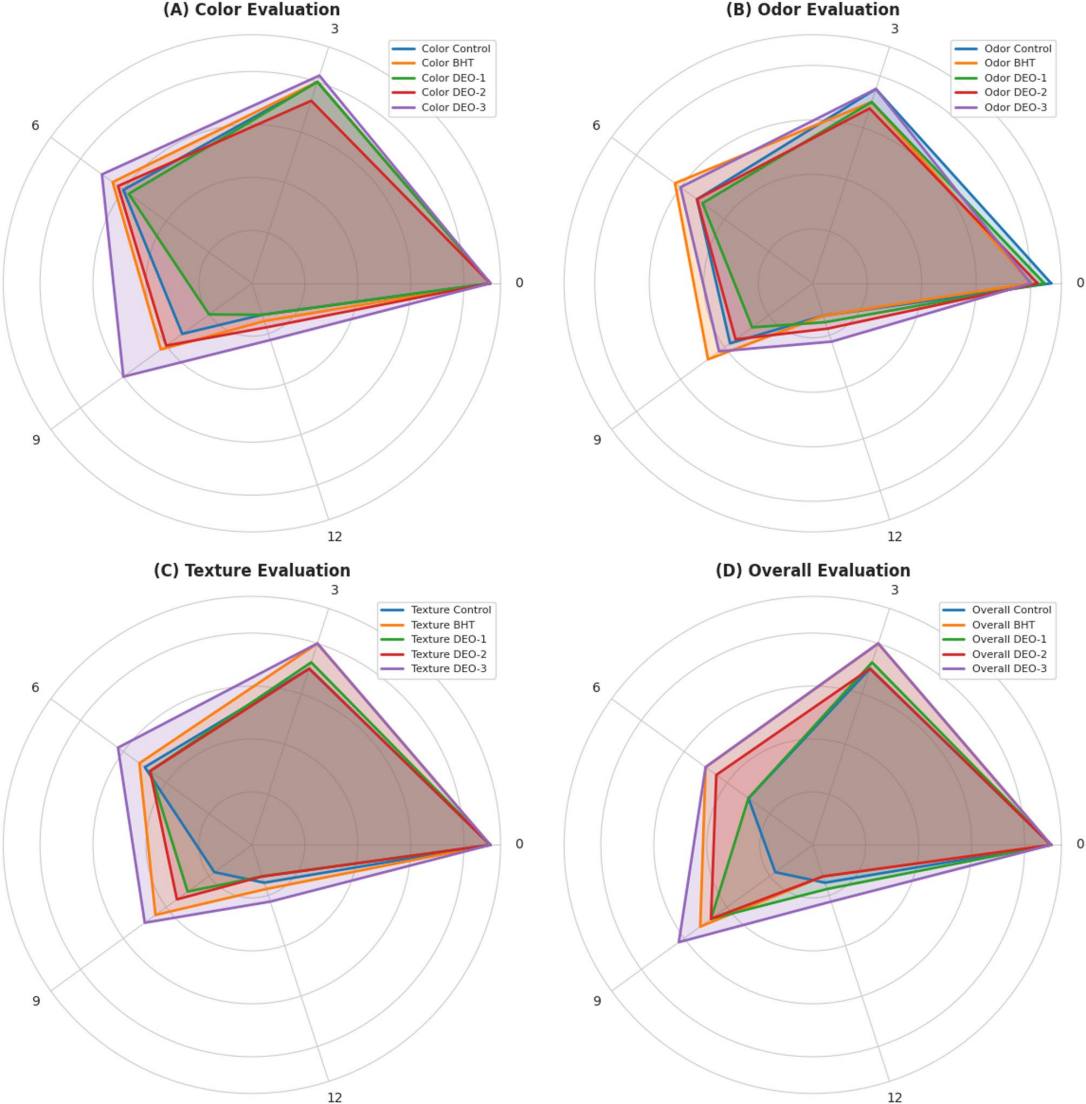
Sensory evaluation of shrimp meat treated with different concentrations of Dill seed essential oil compared to BHT and a control group over a 12-day storage period. The sensory attributes assessed included color (**A**), odor (**B**), texture (**C**), and overall acceptability (**D**) at different time intervals.

As storage time increased, all samples experienced progressive deterioration in sensory characteristics, including color, aroma, and overall acceptability, as evaluated using the 9-point hedonic scale (Fig. 8). This decline was particularly noticeable in the untreated control group. The figure shows a substantial decrease (*p* < 0.05) in organoleptic scores across all shrimp samples throughout the 12-day storage period at 2.5 ± 0.5 °C. After refrigerated storage, the sensory evaluation scores diminished for all samples. The *DSEO* emulsion and BHT treatment groups exhibited the least reduction in scores, whereas the control samples showed the greatest decrease in scores.

**Fig. 9:**
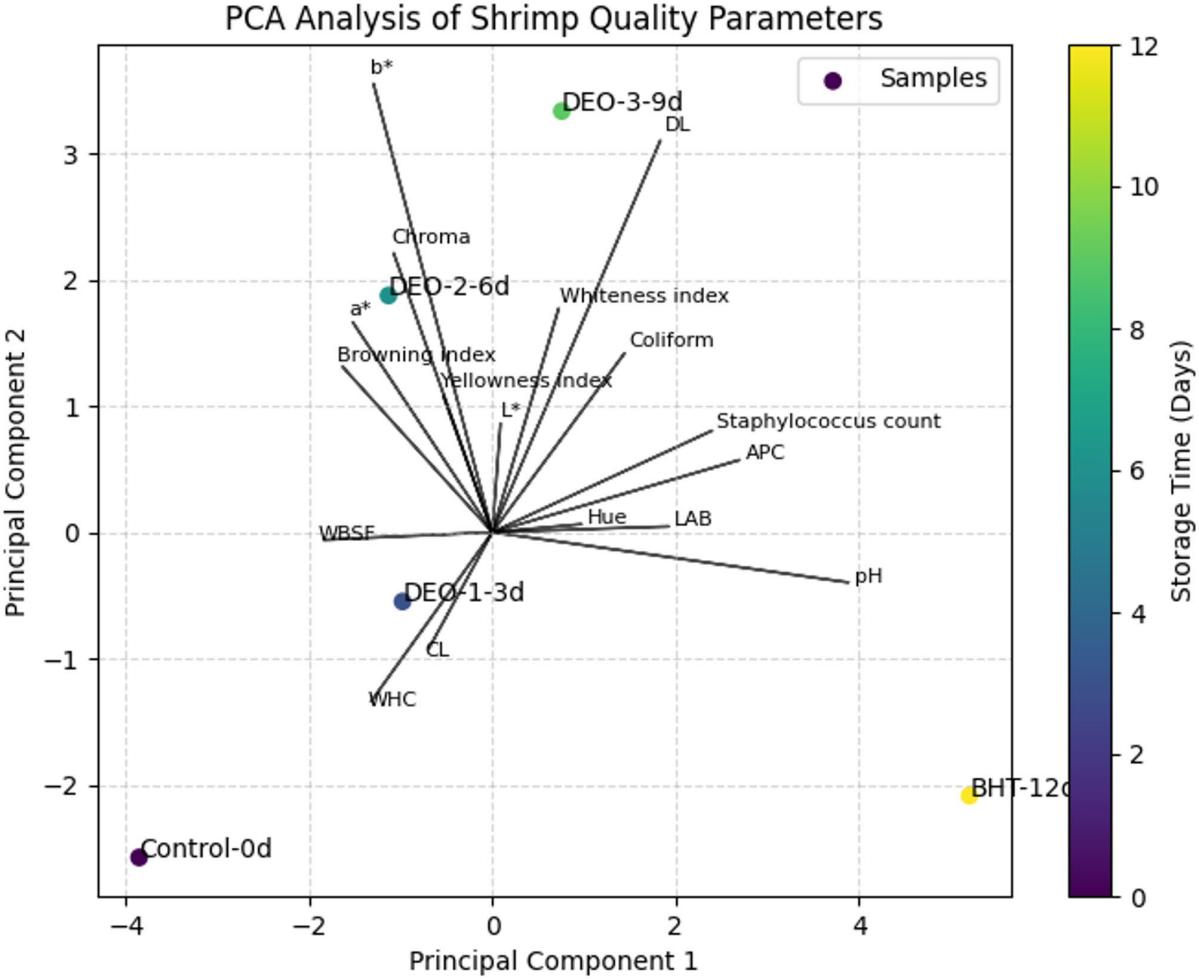
PCA analysis of treatment effects.

Principal Component Analysis (PCA) was used to assess the impact of dill seed essential oil emulsion (*DSEO*) concentrations and butylated hydroxytoluene (BHT) on the microbiological, physicochemical, and instrumental color attributes of refrigerated shrimp meat over 12 days (Fig. 9). This approach visualizes the relationships among parameters, identifies influential variables, and compares treatment effects. The PCA biplot provides an overview of shrimp quality parameter variance across treatment groups (Control, *DSEO*-1, *DSEO*-2, *DSEO*-3, and BHT) and storage durations (0, 3, 6, 9, and 12 days). The first two principal components (PC1 and PC2) accounted for the majority of the variance. PC1 captured variations in microbiological indicators and physicochemical properties, whereas PC2 explained variations in instrumental color parameters. The directional vectors indicate the contribution of each parameter to the observed variance and the correlation. The pH vector had a prominent influence on PC1, whereas the color indices were more strongly associated with PC2. Vector length and orientation illustrate the influence of the storage duration and treatment on specific parameters. The microbial counts increased over time, particularly in the control group, indicating shrimp spoilage. The *DSEO*-treated samples exhibited separation in the PCA space, indicating reduced microbial growth and improved preservation of the physicochemical properties and color stability. PCA results highlighted that dill seed essential oil preserved shrimp quality during refrigerated storage, with a stronger influence on microbial inhibition and color retention than the control group. The BHT-treated samples also demonstrated a significant separation from the control. The clustering patterns suggested that higher *DSEO* emulsion concentrations (*DSEO*-2 and *DSEO*-3) provided more pronounced preservation effects, positioning these treatments as promising alternatives to synthetic antioxidants in seafood storage.

## Discussion

Dill seed essential oil (*DSEO*) primarily consists of monoterpenes, with key components including α-phellandrene, d-limonene, carvone, and dill ether. These findings align with those of Kostić et al.^41^, who identified carvone (42.47%), limonene (29.04%), and α-phellandrene (13.12%) as predominant constituents. Similarly, Benlembarek et al.^42^ found that carvone (34.33%), α-phellandrene (22.03%), diethyl ether (18.84%), limonene (6.93%), and dill apiol (5.01%) were significant components^43,44^further confirming that carvone and limonene are the most common monoterpenes. However, El-Sayed et al.^45^ reported a different composition, with dillapiole (44.01%), d-limonene (19.47%), and carvotanacetone (14.03%) being the major elements. Variability in concentration and composition is influenced by factors such as climate, geographic location, metabolic processes, maturity, and the plant part used for extraction^46^. Additionally, factors like harvesting time, storage conditions, and extraction methods can impact the yield and composition of essential oils^47^.

Regarding total phenolic content (TPC) and total flavonoid content (TFC), this study found significant concentrations, comparable to the findings of Hadi et al.^48^, who reported that ethanolic extracts of *Anethum graveolens* contained high levels of phenolic compounds and flavonoids at approximately 52.65 ± 0.22 mg GAE/g of extract and 35.58 ± 2.79 mg QE/g of extract. Flavonoids and phenolic compounds exhibit strong antioxidant properties^49^with antioxidant activity primarily attributed to phenolic compounds. These compounds help neutralize free radicals, quench reactive oxygen species, and break down peroxides^50^. A higher concentration of phenolic compounds enhances hydrogen donation to free radicals, increasing inhibitory strength^51^. Thus, due to its high phenolic content, *DSEO* is considered a promising natural antioxidant. DPPH radical scavenging assays are widely used to assess antioxidant capacity due to their stability^52^. The current study DPPH assay showed that *A. graveolens* essential oil displayed concentration-dependent inhibition of DPPH radicals. The antioxidant potential of *DSEO* may be linked to its monoterpenes, which function as radical scavengers. Essential oils with monoterpene hydrocarbons, oxygenated monoterpenes, and sesquiterpenes typically demonstrate strong antioxidant activity^53^. Limonene, a key component in *DEO*, is recognized as a generally safe (GRAS) antioxidant for human consumption by the Code of Federal Regulations (Sun, 2007). Moreover, limonene, classified as a monoterpene phenol, exhibits antioxidant properties^54^.

Postmortem alterations in shrimp and degradation of muscle proteins during storage can be reflected in pH changes^55^. All shrimp groups experienced a gradual pH increase over the storage period, with the control group exhibiting the highest increase. *DSEO*-3 effectively inhibited pH rising due to its antimicrobial properties, protein preservation capabilities, and reduction of compounds responsible for pH elevation, consistent with previous research demonstrating *DEO*’s antimicrobial potential^44,56^. The accumulation of basic compounds from bacterial or enzymatic activity contributes to pH changes in shrimp^57^. The results suggest that *DSEO* treatment helps maintain shrimp quality by slowing spoilage. *DSEO*-treated shrimp retained higher water-holding capacity (WHC) than both the control and BHT-treated groups up to day 9, except on days 6 and 12. WHC was highest in BHT-treated samples on day 6, while BHT and *DSEO*-2 groups maintained elevated WHC on day 12. Protein denaturation, caused by enhanced microbial activity and metabolic processes, especially under high pH circumstances, leads to a decrease in WHC^58^. The WHC reduction in control shrimp is likely due to protein denaturation during storage. *DSEO*-treated groups maintained higher WHC, indicating structural protein stability attributed to *DSEO*’s antioxidant activity, mainly from carvone and limonene. Protein degradation, denaturation, or oxidation leads to weight loss due to muscle drip loss^59^. Since weight retention is a critical factor in evaluating preservative effectiveness, reduced drip loss in all *DSEO*- and BHT-treated shrimp samples, except *DSEO*-3 on day 3, suggests effective preservation. The lowest drip loss observed in *DSEO*-3 samples indicates *DSEO*’s role in preventing protein degradation. Similar trends have been noted by Qian et al.^60^, who reported that essential oils minimized drip loss in Pacific white shrimp stored at low temperatures.

*DSEO*-1 and *DSEO*-3 exhibited lower cooking loss (CL) than the control throughout storage, whereas shrimp treated with BHT, and *DSEO*-2 demonstrated higher CL, except on day 3. Elevated CL is indicative of lipid and protein oxidation, which compromises muscle WHC. Research suggests that synthetic antioxidants such as BHT mainly counter lipid oxidation but are less effective against protein oxidation, a crucial factor for WHC and CL^61^. The ability of *DSEO*-1 and *DSEO*-3 to reduce CL suggests dill essential oil’s effectiveness in preserving shrimp moisture and mitigating cooking loss due to protein oxidation, which affects consumer acceptability. Postmortem pH decline and muscle protein reorganization also contribute to these changes. The primary muscle components responsible for shrimp texture variations include myofibrillar and connective proteins^62^.

Differences in Warner-Bratzler shear force (WBSF) values across treatment groups likely stem from the interaction between postmortem proteolysis and antioxidative properties. The control group exhibited a steady decline in WBSF over time, indicative of muscle softening caused by endogenous proteases, such as calpains and cathepsins, which degrade myofibrillar proteins^63,64^. *DSEO*-3 treatment resulted in a progressive WBSF increase until day 9, followed by a decline on day 12. Meanwhile, BHT, *DSEO*-1, and *DSEO*-2 groups showed a general decrease in WBSF, except on day 6 for BHT and day 3 for *DSEO*-2. These findings suggest that *DSEO* stabilizes muscle proteins due to its antioxidative constituents, particularly carvone and limonene, which neutralize reactive oxygen species (ROS), preventing oxidative damage to essential muscle proteins such as actin and myosin^44,65^. *DSEO* may also form hydrogen bonds or hydrophobic interactions with myofibrillar proteins, enhancing their conformational stability^44,60,66^. However, as bioactive components degrade during prolonged storage, microbial activity accelerates proteolysis, leading to muscle weakening^44,67,68^.

The first qualitative feature of food, usually noticed by consumers, is its appearance and color. Therefore, color is one of the most substantial and prominent aspects of food that affects consumer acceptance. These results indicated that the color properties of shrimp were significantly influenced by *DSEO*. The lightness (L*) of treated samples increased considerably during storage. BHT and other *DSEO-*treatments maintained lightness better, perhaps due to their antioxidative qualities that limit pigment breakdown. The redness coordinates (a*) of all treated samples, primarily BHT and *DSEO*, exhibited decreased values compared to the control group throughout the storage period, with the exception of *DSEO*-2, which displayed a greater a* value than the control on day 9. The elevation of redness value may be associated with the degradation of astaxanthin-protein complexes; astaxanthin (AST) is a carotenoid found in the shells of crustaceans, leading to the liberation of astaxanthin monomers^69^. However, the yellowness (b*) coordinate displayed a higher value in both BHT and *DSEO*-1, especially at D12, compared to the control, but *DSEO*-2 and *DSEO*-3 showed higher b* values at the end of the storage period (D9 and D12) relative to the control. By Day 12, all treated groups maintained higher b* values than the control group, highlighting their ability to preserve yellowness. The whiteness value of all groups increased during storage, with the exception of *DSEO*-1, which showed a decreasing trend. However, the control group showed no distinctive pattern, and the BHT-treated group showed a decreasing value at D9. All treated shrimps, primarily BHT and *DSEO*, showed higher whiteness levels than the control, suggesting a more desirable commercial appeal. This suggests that both BHT and *DSEO* have anti-melanotic effects. The enhanced antioxidant activity of *DSEO* helped retain the whiteness of shrimp. Similar findings were reported by Qian et al.^70^who reported that shrimp treated with essential oils, primarily oregano essential oil and clove leaf essential oil, had higher whiteness values than the control. The ΔE values, an indicator of the total color difference, exhibited no discernible trend across the treated groups, especially the BHT and *DSEO*-1and *DSEO*-2 treated groups during the storage period, with the *DSEO*-3 treated group displaying the lowest color change. These color changes are probably attributed to the oxidation of lipids during the breakdown of carotenoids by endogenous enzymes and their release from the protein matrix in the muscle^71^.

The use of dill seed essential oil markedly improved the microbiological quality of shrimp, attaining an acceptable level below six logs for APC by day 9, with the exception of *DSEO*-1, whereas the control samples surpassed these limits sooner. A comparable trend was observed for *DSEO*, specifically *DSEO*-3, concerning both *LAB* and coliform counts. Additionally, the staphylococcal counts of all samples treated with *DSEO* showed a significant decrease on day 12 compared to those treated with BHT and the control. These findings are in accordance with those of Caglak and Karsli (2023)^72^who investigated the effect of dill extracts on the microbiological quality of rainbow trout croquettes. These results are ascribed to the antibacterial properties of *DEO*. Several studies have emphasized these actions^23,44,72–74^. Earlier studies pointed out that the primary chemical profile of *DEO* is characterized by carvon and D-limonene, which are both potent natural antimicrobial substances. In general, the antibacterial activity of EO compounds is influenced by their hydrophobicity, stability, and volatility. For example, limonene is highly volatile, prone to oxidation, and poorly soluble in water^75^. Consequently, a high concentration of d-limonene may not result in a significant antibacterial effect^76^. In the current study, *LAB* were not substantially influenced by the application of *DSEO*, which may be attributed to the generally lower inhibitory effect of EO on gram-positive bacteria, such as *lactic acid bacteria*, because of the unique characteristics of their cell walls that provide resistance to specific antimicrobial agents^77^. The mechanism of D-limonene resembles lactobacilli in relation to antibiotics, wherein D-alanine, the terminal amino acid in the peptidoglycan layer of the bacterial cell wall, is substituted with D-lactate or D-serine, thereby obstructing the binding of the antimicrobial agent to the peptide chain and resulting in the suppression of these bacteria^76,78^.

To a certain extent, sensory evaluation reflects consumers’ preferences for specific products and the overall quality of products. The alterations in the sensory characteristics (texture, aroma, hue, and overall palatability) of all shrimp samples following refrigerated storage were evaluated using a 9-point hedonic scale. Scores under 7 are considered inadequate for customers^14^. Decline in organoleptic ratings for all shrimp samples throughout a 12-day storage period at 2.5 ± 0.5 °C was presumably attributable to microbial activity and chemical alterations. Minimal organoleptic properties were observed for the *DSEO*-fortified groups. Thus, the inclusion of *DSEO*, owing to its antioxidant and antibacterial properties, may have a protective effect against chemical and microbial alterations, thereby alleviating the negative impacts on sensory characteristics.

## Conclusion

This study investigated the preservative effects of *Anethum graveolens* essential oil (*DSEO*) emulsion on shrimp (*Litopenaeus vannamei*) during refrigerated storage, and examined its physicochemical, microbiological, and sensory attributes. *DSEO* emulsion treatment significantly influenced the physicochemical parameters, with *DSEO*-3 showing the lowest pH and drip loss values. *DSEO* emulsion improved the microbiological quality of shrimp, with *DSEO*-3 achieving acceptable levels of aerobic bacteria, lactic acid bacteria, coliforms, and staphylococcal counts. The sensory attributes declined in all samples during storage, with minimal reductions in the *DSEO*-treated groups. *DSEO* emulsion has demonstrated potential as a natural preservative for extending shelf life and maintaining shrimp quality while inhibiting foodborne pathogens.

## Data Availability

All data generated during the current study are available from the corresponding authors on reasonable request.
